# A Rare Case of High-Pressure Paint Injection Causing a Penetrating Neck Injury

**DOI:** 10.7759/cureus.75280

**Published:** 2024-12-07

**Authors:** Daniel Saad, Aaditya Dravid, Issa Beegun

**Affiliations:** 1 Otolaryngology, Imperial College London Healthcare National Health Service (NHS) Trust, London, GBR; 2 School of Medicine, Imperial College London Healthcare National Health Service (NHS) Trust, London, GBR

**Keywords:** ent emergency, high-pressure injection injuries, major trauma, otolaryngology case report, paint, paint industry workers, penetrating trauma neck

## Abstract

We report a case of a 45-year-old gentleman who presented to our major trauma centre after sustaining a penetrating high-pressure paint injection injury to the neck. This rare mechanism of injury is most commonly reported to affect the non-dominant hand, occurring due to the malfunction or misuse of industrial paint machines, causing a piercing soft tissue injury with high-pressure fluid. The unique challenges faced in managing penetrating injuries to the neck are due to the density of vital visceral structures in the region, including major blood vessels and the upper aerodigestive tract. Whilst there is one documented case of a water injection injury, there are no published reports involving paint injection injuries to the neck at the time of writing this report. As such, we present this unique case to highlight important learning points and the challenges faced in our experience of managing a penetrating paint injury of the neck at our major trauma centre.

## Introduction

High-pressure injection injuries (HPII) caused by paint have been reported in the scientific literature since 1967, after the commercialisation of industrial high-pressure painting machines [1]. The upper limb extremity is most commonly affected, with the largest published systematic review identifying 435 cases between 1966 and 2006 [2]. Other rarely reported anatomical areas affected by paint-induced HPII include the face and orbits [3-5]. There are no reported cases of pain-induced HPII in the neck to date. However, a water injection injury to the neck has been reported that was managed non-operatively [6].

Vital neurovascular and visceral structures in the neck are bundled in deep cervical fascial compartments below the platysma muscle. A penetrating neck injury is defined as a wound that breaches the platysma, thus risking damage to deeper structures. This is a major trauma event, carrying a high mortality rate of up to 10%, and as such, should be initially managed with Advanced Trauma Life Support (ATLS) principles [7]. Unstable patients should be taken for immediate surgical exploration, whereas stable patients should be investigated with contrast-enhanced CT angiography to assess the extent of injury [8].

Herein, we report a case of an extensive paint HPII to the anterior triangle of the neck in a 45-year-old man who presented to our major trauma centre. We highlight important learning points in the surgical management of the initial penetrating injury and the sequelae of the high-pressure paint injection in the neck.

## Case presentation

**Figure 1:**
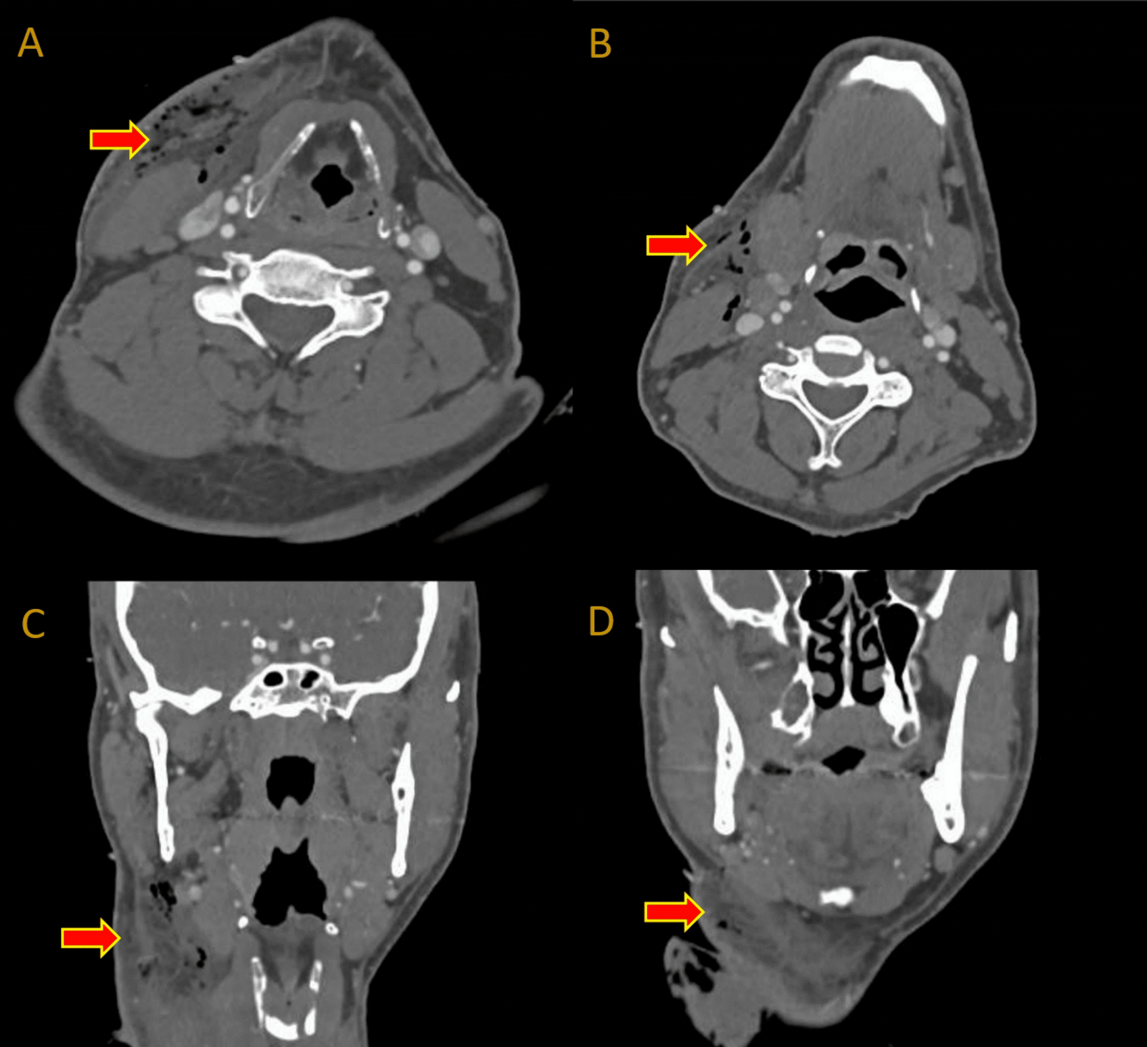
A preoperative contrast-enhanced computed tomography scan of the neck. Axial slices (panes A and B) and coronal slices (panes C and D) with arrows highlighting areas of surgical emphysema, disorganised haematoma, and paint infiltration of superficial and deep cervical tissue planes.

**Figure 2:**
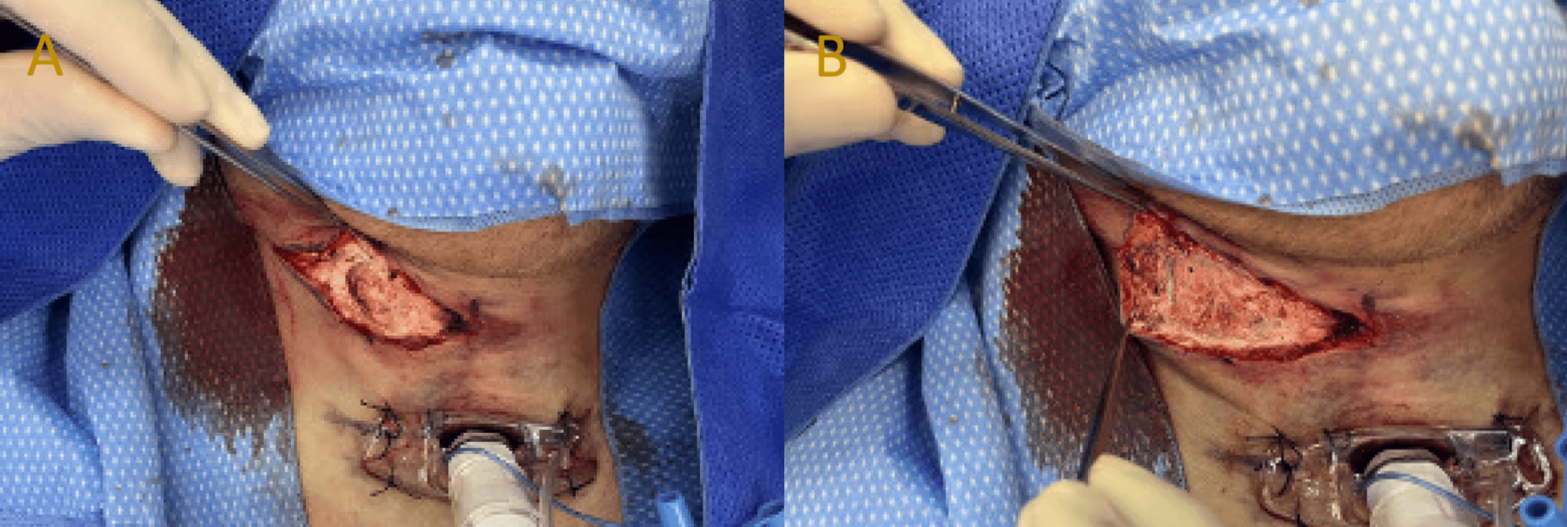
Intraoperative photographs displaying the site of neck injury and paint contamination of the superficial cervical fascia and subcutaneous tissue.

A 45-year-old painter was brought by ambulance to our major trauma centre one hour after sustaining a penetrating anterolateral neck injury of an unclear mechanism. He presented with a 2 cm right-sided, zone 1 neck wound, a hoarse voice, and a palpable haematoma. The patient was immediately intubated to secure the airway, and a contrast-enhanced computed tomography (CT) scan of the neck (Figure 1) was obtained. The CT neck reported an extensive, disorganised subcutaneous haematoma of the right anterolateral neck without an active bleeding point. The patient was taken to the operating theatre for a primary exploration and evacuation of the suspected haematoma. Surgical exploration revealed copious amounts of white-coloured paint throughout multiple superficial and deep fascial compartments (Figure 2). The paint had infiltrated the surrounding soft tissues of the anterolateral neck and penetrated through the platysma to reach deep cervical musculature. In the absence of intraoperative findings of any significant bleeding or haematoma, it became clear that the CT scan findings were indicative of the paint infiltrate rather than a haemorrhagic process.

Wound exploration and oesophagoscopy excluded any serious injury to vital structures in the neck, but there was great difficulty in removing the paint from the surgical field. Attempts at irrigating the wound with Betadine (10% povidone-iodine) and warmed sodium chloride solution were unsuccessful. A brief intraoperative literature search for similar cases was unhelpful; hence the airway was secured with a tracheostomy and the wound primarily closed with a neck drain in situ. A nasogastric tube was also sited to allow enteral feeding during the recovery period. The patient was treated post-operatively in the intensive care unit (ICU) for four days and returned to the operating theatre for further wound exploration and debridement of the remaining paint material. Intraoperatively, the paint-contaminated soft tissue was found to have developed interval fibrosis. This included subcutaneous fat and segments of cervical musculature (sternocleidomastoid and infrahyoid strap muscles). The fibrotic, paint-contaminated structures were debrided with monopolar diathermy, and the wound was primarily closed with a Penrose surgical drain in situ.

The patient was treated with a course of intravenous broad-spectrum antibiotics and high-dose intravenous corticosteroids as adjuncts to surgical management. The patient’s tracheostomy was successfully decannulated on the seventh day of admission, and he swiftly recovered until discharge on the eleventh day of admission. Fortunately, his swallow and speech functions were not significantly affected by the injury or surgical interventions. He was able to tolerate an oral diet in the early postoperative period and used a speaking valve while the tracheostomy was in situ. A follow-up appointment two weeks after discharge revealed he was managing well at home and had recovered with a full range of motion in the neck and retained normal swallow and speech functions.

## Discussion

The severity of HPII may initially seem limited; however, caustic substances (such as paint) within human tissue lead to an inflammatory cascade with histological effects including neutrophilic inflammation, tissue oedema, and necrosis [9,10]. This then leads to sequelae such as neurovascular injury, infection, compartment syndrome, and even amputation. Hence, early treatments, including debridement, irrigation, compartment decompression, and antibiotic therapies, are paramount to improving outcomes [11-13].

In experimental studies, it is reported that human skin can be breached by only 100 psi of pressure [14]. In this case, an industrial paint spray gun was used at approximately 3000 psi. Due to the immense pressures involved, significant volumes of paint can pass through a small entry point in the skin to spread extensively through multiple fascial compartments. Hence, in conjunction with radiological imaging, a thorough surgical exploration is warranted to identify the extent of the injury and contamination.

Patients with injection injuries to the hand often present late or are initially dismissed by healthcare professionals due to the delayed onset of significant symptoms and signs, leading to delayed treatment, which affects outcomes. In contrast, penetrating neck wounds often present emergently with minimal delays to surgical intervention. In our case, the patient was taken to the operating room within 12 hours of the injury. With this limited contact time, the paint had not yet significantly reacted with the surrounding tissues. This posed a major operative challenge as the paint could neither be washed out nor easily debrided from the tissues. A second exploratory operation was therefore essential to debride the paint and affected fibrotic tissues, reducing the risk of complications such as scarring and contracture.

Although relatively rare, the clinical importance of paint-induced HPII lies in the associated high morbidity rates, including high reported digit amputation rates between 0-48%, depending on the material injected and the anatomical location [2,14,15]. Industry safety standards include the use of gloves and eye protection, which would reduce the risk of skin irritation and eye contamination, respectively, but do not offer significant protection against penetrating injury.

## Conclusions

In summary, this is a rare presentation of a penetrating neck injury caused by high-pressure paint injection, which has never before been reported in the literature. Extracting the paint from the wound presented a unique surgical dilemma as it was not amenable to irrigation. Surgical debridement was only possible after allowing the paint to react over four days from the initial injection. The resultant inflammatory reaction formed fibrotic tissue, which could be identified and dissected from healthy tissue. This would not have been possible to perform during the primary exploration.

As with any penetrating neck injury, we recommend HPIIs of the neck be managed according to ATLS principles, which prioritise stabilising the patient and securing the airway before considering washout and debridement. Broad-spectrum antibiotics should be used to prevent operative site infections, and corticosteroids should be considered to minimise the risk of swelling around the airway.
